# Coq4 deficiency induces placental vascular development defects through FSP1/CoQ10 axis-mediated endothelial ferroptosis

**DOI:** 10.3389/fcell.2026.1774201

**Published:** 2026-03-02

**Authors:** Rui Chen, Sikun Wang, Xueke He, Zhanbiao Wang, Miao Jiang

**Affiliations:** 1 Institute of Cardiovascular Disease, Key Laboratory for Arteriosclerology of Hunan Province, Hunan International Scientific and Technological Cooperation Base of Arteriosclerotic Disease, Hengyang Medical School, University of South China, Hengyang, Hunan, China; 2 Institute of Pathology and Southwest Cancer Center, Southwest Hospital, Third Military Medical University (Amy Medical University), and The Key Laboratory of Tumor Immunopathology, The Ministry of Education of China, Chongqing, China

**Keywords:** placental vascular development, ferroptosis, CoQ10, Coq4, FSP1

## Abstract

**Introduction:**

Coenzyme Q10 (CoQ10), a critical electron carrier in mitochondrial respiratory chains, is essential for cellular energy metabolism. Ubiquinone biosynthesis protein 4 homolog (Coq4), a rate-limiting enzyme in CoQ10 biosynthesis, is indispensable for embryonic development. However, the mechanisms underlying Coq4 deficiency-induced developmental defects remain elusive. Emerging evidence highlights the FSP1/CoQ10 axis as a central regulator of lipid peroxidation and ferroptosis, a non-apoptotic cell death mechanism implicated in placental vascular dysgenesis and trophoblast dysfunction. This study aims to elucidate the molecular mechanisms by which Coq4 deficiency disrupts placental development, with a focus on the interplay between the FSP1/CoQ10 axis and endothelial ferroptosis.

**Methods:**

Coq4^+/−^ mice were generated via CRISPR-Cas9-mediated genome editing. Offspring were genotyped by Polymerase Chain Reaction (PCR), and placental tissues were collected at E9.5 for histological analysis and immunofluorescence. Lentivirus-mediated Coq4 knockdown in human umbilical vein endothelial cells (HUVECs) was combined with RNA sequencing (RNA-seq) to identify differentially expressed genes. Key pathway proteins were validated by Western blotting.

**Results:**

Coq4^−/−^ embryos exhibited embryonic lethality and the placentas showed vascular rarefaction and impaired trophoblast invasion. Transcriptomic profiling and Western blotting identified upregulated ferroptosis-related genes including acyl-CoA synthase long-chain family member 4 (ACSL4), ferritin heavy chain 1(FTH1) and downregulated Ferroptosis Suppressor Protein 1(FSP1), but without changes observed on the glutathione peroxidase 4 (GPX4). FSP1 overexpression or CoQ10 supplementation alone partially alleviates ferroptosis whereas combined intervention more effectively improves it.

**Discussion:**

This study demonstrates that Coq4 deficiency induces endothelial ferroptosis via disrupting the FSP1-CoQ10 antioxidant axis, and may also provide new insights into the pathogenesis of pregnancy complications caused by placental dysfunction and iron-related vascular diseases, while offering novel approaches for exploring potential therapeutic targets.

## 1 Introduction

Coenzyme Q Biosynthesis Protein 4 Homolog (Coq4) is located at position 34.11 on human chromosome 9, encoding the Coq4 protein (Xie et al., 2022). It is widely expressed in human tissues and serves as a key factor in the biosynthesis of coenzyme Q10 (CoQ10) in both yeast and human cells (Casarin et al., 2008; Alcázar-Fabra et al., 2021). CoQ10 is a lipid molecule composed of a benzoquinone ring and a diene-like side chain, widely distributed in all eukaryotic cells (Arenas-Jal et al., 2020). Beyond its role as an electron carrier in the mitochondrial respiratory chain, it exhibits anti-inflammatory and antioxidant functions (Mantle et al., 2023) and may participate in regulating cellular processes such as proliferation and apoptosis (Fontaine et al., 1998). Current research on Coq4 primarily focuses on the clinical syndromes caused by its mutations. Studies have revealed that autosomal recessive Coq4 mutations lead to multisystem mitochondrial disorders, particularly affecting the central nervous system when primary CoQ10 synthesis is deficient (Mero et al., 2021; Cordts et al., 2022; Bosch et al., 2018). The precise biochemical synthesis pathway of CoQ10 in mammals remains incompletely understood. Recent studies have revealed that CoQ4 catalyzes the decarboxylation of the C1 position on the CoQ10 aromatic ring, filling a critical gap in its biochemical mechanism (Pelosi et al., 2024). Notably, studies have identified CoQ4 expression loss or dysfunction in placental tissues with impaired embryonic development, and CoQ4 gene mutations have been detected in CoQ10 deficiency patients (Perez-Garcia et al., 2018). This suggests Coq4 may play a crucial role in embryonic development or placental formation.

Ferroptosis is an iron-dependent, regulated cell death characterized by the massive accumulation of lipid peroxides. It fundamentally differs from traditional apoptosis and necrosis in cellular morphology, biochemical markers, and molecular mechanisms. This process involves the synergistic dysregulation of three core mechanisms: iron metabolism imbalance, uncontrolled lipid peroxidation, and exhaustion of endogenous antioxidant systems (Tang et al., 2021). Within the cellular defense system against ferroptosis, the glutathione peroxidase 4 (GPX4) pathway is recognized as the dominant regulatory axis. This pathway utilizes glutathione (GSH) as substrates to specifically catalyze the reduction of peroxidized lipids into non-toxic lipid derivatives, thereby directly suppressing the chain reaction of lipid peroxidation and inhibiting ferroptosis (Stockwell et al., 2017). Beyond the canonical GPX4 pathway, it has uncovered a parallel and functionally independent defense axis—the FSP1 pathway (Hussain et al., 2024; Ma et al., 2025a). Kirill Bersuker and colleagues demonstrated that FSP1, initially termed apoptosis-inducing factor mitochondrial 2 (AIFM2), acts as a coenzyme Q10 reductase in suppression of iron-dependent lipid damage, with both pathways operating independently to maintain redox homeostasis. Even when GPX4 retains its enzymatic activity, FSP1 deficiency alone is sufficient to induce ferroptosis, confirming the autonomy of this pathway (Bersuker et al., 2019; Doll et al., 2019). FSP1 orchestrates cellular redox homeostasis primarily through the FSP1-CoQ10-NAD(P)H antioxidant axis, where it neutralizes lipid peroxides, thereby exerting its anti-ferroptosis function (Doll et al., 2019). The expression and enzymatic activity of FSP1 are regulated by transcription factors, non-coding RNAs and post-translational modifications including acetylation and methylation (Li et al., 2023).

The placenta plays an indispensable central role in fetal development prior to birth. Abnormalities in its structure and function can lead to various pregnancy complications such as preeclampsia, fetal growth restriction, and miscarriage (Santos et al., 2026). The mature placenta primarily consists of three components: the outer maternal portion, including uterine decidual tissue and the uterine vascular system responsible for maternal blood perfusion; the junctional zone, which anchors the fetal placenta to the uterine wall and contains trophoblast cells capable of invading maternal uterine tissue and blood vessels; and the labyrinth zone, composed of highly branched villi that serve as the critical site for efficient maternal-fetal exchange of substances. The villi float within the maternal bloodstream. Their structure incorporates an outer epithelium, mesenchymal cells, and vascular endothelial cells derived from the trophoblast cell lineage. Together, these elements mediate the complex interactions between fetal and maternal tissues (Hemberger et al., 2020; Knöfler et al., 2019; Xiao et al., 2020). The normal development of placental blood vessels provides the structural foundation for ensuring adequate blood flow, oxygen, and nutrient supply to the fetus, playing a crucial role in sustaining fetal growth and development (Aplin et al., 2020; Jiang et al., 2025). Vascular development primarily involves two key stages: angiogenesis and neovascularization, which encompass the differentiation and proliferation of endothelial cells and the remodeling of vascular networks (Furtado and Eichmann, 2024). Angiogenesis occurs predominantly during early embryonic development, where endothelial progenitor cells differentiate, expand, and fuse to form a preliminary vascular network. Upon this foundation, complex and functionally mature vascular networks gradually emerge through neovascularization (Majesky, 2018; Carmeliet and Jain, 2011). Angiogenesis is a highly intricate biological process involving the activation, proliferation, and migration of vascular endothelial cells. It is precisely regulated by multiple growth factors and signaling pathways, such as the vascular endothelial growth factor (VEGF) and its receptor pathways (VEGF/VEGFR) (Dalal et al., 2025; Yang et al., 2022). Simultaneously, vascular endothelial cells serve not only as the vascular barrier but also as the regulatory hub for vascular homeostasis, participating in multiple critical physiological processes including vascular tone, permeability, immune responses, hemostasis, repair, and metabolism (Xu et al., 2021). Therefore, the structural and functional integrity of vascular endothelial cells is a key factor in the normal development of placental vasculature. Any form of dysfunction or injury to these cells may lead to abnormal vascular formation, subsequently affecting placental function and pregnancy outcomes.

Studies have demonstrated that ferroptosis plays a pivotal role in the pathogenesis of diverse diseases, including atherosclerosis (Zhang et al., 2024), neurodegenerative disorders (Wang et al., 2024), myocardial ischemia/reperfusion injury (Laukaitiene et al., 2023), cancer (Su et al., 2024), and organ system development disorder. Laura H. Steenberge and colleagues observed that exogenous supplementation with Coq4 effectively alleviates ferroptosis progression in CoQ2-deficient HepG2 cells (Steenberge et al., 2024). However, whether ferroptosis contributes to developmental disorders caused by Coq4 deficiency or mutations remains unclear. To address this, our study employed CRISPR-Cas9 technology to generate Coq4 heterozygous knockout mice. After breeding, Coq4 knockout mice were utilized to assess placental vascular development. Additionally, we utilized lentivirus-mediated stable knockdown of Coq4 in human umbilical vein endothelial cells (HUVECs) and employed RNA-sequencing, Western blotting analysis, and transmission electron microscopy (TEM) to investigate the mechanisms underlying Coq4 deletion-induced placental vascular developmental defects.

## 2 Materials and methods

### 2.1 Cell culture and CoQ10 treatment

HUVECs were purchased from Cell Bank at the Chinese Academy of Sciences. Cells were routinely cultured in DMEM medium (Gibco, REF: C11995500BT) supplemented with 10% fetal bovine serum (ExCell Bio, Cat: FSP500) at 37 °C in a humidified incubator with 5% CO_2_.

CoQ10 was purchased from MedChemExpress (Cat: HY-N0111). To prepare the CoQ10 stock solution, 10 mg of CoQ10 powder was precisely weighed and dissolved in 1.1583 mL of N, N-dimethylformamide (DMF) (MCE, Cat: HY-Y0345) using a 37 °C water bath and 40 kHz ultrasonication for 10 min to prepare a 20 mM stock solution. When the growth density of the cells to be treated reached 70%–80%, CoQ10-containing culture medium was added, and cells were maintained for 48 h for subsequent experiments.

### 2.2 Western blotting

The RIPA lysis buffer (Epizyme, Cat: PC101) was mixed with protease inhibitor (Abiowell, Cat: AWB0159a) at a ratio of 100:1 and added to cells rinsed with PBS. After thoroughly lysis on ice for 30 min, the lysate was centrifugated at 4 °C, 12,000 rpm for 10 min and the supernatant was collected as the total protein sample. Protein samples were mixed with 5× loading buffer (Cwbio, Cat: CW0027S) at a 4:1 ratio and heated in a 95 °C metal bath for 6–8 min to denature proteins. Equal amounts of protein were separated by SDS-PAGE and electrotransferred to a PVDF membrane (Merck Millipore, Cat: IPVH00010). After transferring, the membrane was blocked at room temperature for 1–2 h with TBST containing 5% skim milk powder, then incubated overnight at 4 °C with the corresponding primary antibody. Primary antibody details are as follows: Coq4 antibody (0.04–0.4 μg/mL, Abcam, Cat: ab126295), 4-Hydroxynonenal antibody (1:2000, Bioss, Cat: BS-6313R), ACSL4 antibody (1:5000, Zenbio, Cat: R24265), SLC7A11 antibody (1:1000, CST, Cat: 98051S), FTH1 antibody (1:1000, Zenbio, Cat: R23306), FSP1 antibody (1:8000, Proteintech, Cat: 20886-1-AP), GPX4 antibody (1:2000, Zenbio, Cat: R381958), Beta Actin antibody (1:5000, Servicebio, Cat: GB15003-100), and GAPDH antibody (1:5000, Servicebio, Cat: GB15004-100). The membrane was then incubated with the secondary antibody (1:10,000, Abcam, Cat: ab6721) at room temperature for 1–2 h followed by the addition of enhanced chemiluminescent reagent (Biosharp, Cat: BL520B) and detection on a chemiluminescence imaging system. ImageJ software was used to perform quantitative analysis of the intensity values of the target bands.

### 2.3 Tissue immunofluorescence

Tissue slides were fixed, paraffin-removed, and subjected to heat-induced antigen retrieval. Afterward, the Triple-Label Four-Color Multiplex Fluorescent Staining Kit (Enhanced) (Aifang Bio, Cat: AFIHC034) was employed for subsequent procedures. Specifically, tissue slices were first incubated overnight with the corresponding primary antibody, then incubated at room temperature away from light for 30 min with the kit-provided fluorescently labeled secondary antibody. This step was repeated using another primary antibody from the same species, followed by incubation with the kit-provided secondary antibody labeled with a different wavelength. Finally, sealing the slides with DAPI-containing anti-fluorescence quenching sealing medium, then observing and capturing images under a fluorescence microscope. Primary antibody including Coq4 antibody (1:200, Proteintech, Cat: 16654-1-AP), ACSL4 antibody (1:200, Zenbio, Cat: R24265) and CD34 antibody (1:200, Proteintech, Cat: 14486-1-AP).

### 2.4 Detection of Fe^2+^, GSH, MDA, and CoQ10 content

Intracellular Fe^2+^ levels were measured using the FerroOrange fluorescent probe (Dojindo, Cat: F374). Intracellular glutathione (GSH) content was assessed with the GSH Detection Kit (Nanjing Jiancheng Bioengineering Institute, Cat: A006-2-1). Intracellular malondialdehyde (MDA) levels were measured using the MDA Detection Kit (Beyotime, Cat: S0131S), and intracellular coenzyme Q10 (CoQ10) was detected using the CoQ10 Detection Kit (LEAGENE, Cat: 1227A24).

### 2.5 Construction of Coq4 knockdown endothelial cell lines

Short hairpin RNA (shRNA) targeting the Coq4 gene sequence (shRNA) (GGT​CGC​GAG​TAT​CTC​CGT​TTC) and a negative control (TTC​TCC​GAA​CGT​GTC​ACG​T) were generated and cloned into the GV493 (pFU-GW-016) vector (purchased from Shanghai Genechem) containing a BsmBI site. Recombinant vectors were verified by DNA sequencing. An appropriate volume of viral solution was added to HUVECs (at approximately 20%–30% confluence) using HitransG P infection buffer. After 48–72 h of infection, puromycin was added for 72 h to conduct continuous selection. Subsequently, cells were dissociated into a single-cell suspension and amplified to sufficient numbers, ultimately yielding a stably transfected cell line for subsequent experiments.

### 2.6 Construction of FSP1-overexpressing endothelial cell lines

FSP1 overexpression and control vector (purchased from RIBOBIO, Guangzhou, China) were transfected into Coq4 stably knocked-down endothelial cells at density of approximately 70%–80% using Lipo3000 transfection reagent (Thermo Fisher, REF: L3000015). Subsequent experiments were conducted 48 h post-transfection.

### 2.7 Animal experiments

Coq4 heterozygote mice (Coq4^+/−^) on a C57BL/6 background were purchased from Jiangsu Jicui Yao Kang Biological Co., Ltd. (B6/JGpt⁃ Coq4em2Cd3877/Gpt), which were crossed to generate Coq4 homozygous (Coq4^−/−^) embryos and wild-type (WT) embryos.

All animals used in this study were housed individually in accordance with the Mouse Welfare and Ethics Regulations in University of South China. Housing conditions included a 24-h light-dark cycle (light: 8:00–20:00; dark: 20:00–8:00), ambient temperature of (23 ± 2) °C, relative humidity of 55%–60%, and ad libitum access to food and water. Bedding was changed regularly, and feed and water were replenished as needed. All animal procedures in this study complied with the standards of the Ethics Committee for experimental animals in University of South China.

### 2.8 Statistical analysis

Statistical analysis was performed using Prism 10.0 (GraphPad). Experimental data are presented as mean ± standard deviation. Comparisons between two groups were conducted using t-tests, while comparisons among multiple groups were performed using one-way analysis of variance (ANOVA), Differences were considered statistically significant at *P* < 0.05. Two-way ANOVA was used to assess interactions between two factors, with P < 0.05 indicating statistical significance, the Bliss independence model was employed to evaluate synergistic effects between factors, calculated using the formula: Synergy Score = E_observed_ - E_expected_, A Synergy Score >0 was defined as synergistic interaction.

## 3 Results

### 3.1 Coq4^−/−^ mice exhibit impaired placental vascular development

**FIGURE 1:**
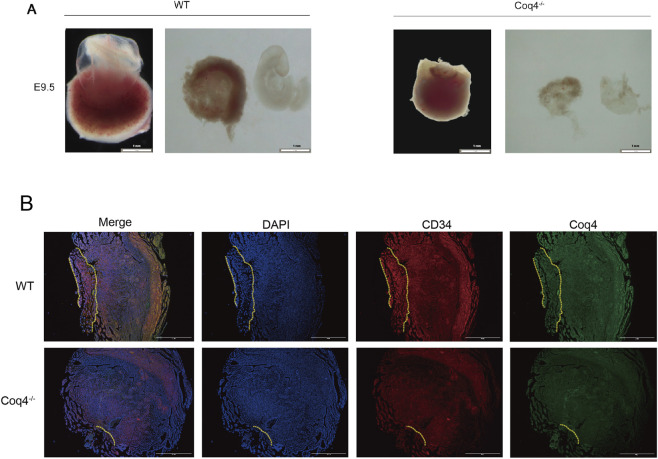
Coq4^−/−^ mice exhibit impaired placental vascular development. **(A)** Light microscopic image of E9.5 placenta (2×),n = 6. **(B)** Immunofluorescence staining of placenta from WT and Coq4^−/−^ mouse with DAPI (blue), CD34 (red), and Coq4 (green). Scale bar = 750 μm, n = 6.

To investigate the physiological function of the Coq4 gene *in vivo*, we successfully generated a Coq4 knockout (Coq4^−/−^) mouse model. Histological analysis revealed that at embryonic day 9.5 (E9.5), angiogenesis in the placenta of Coq4^−/−^ mice was significantly reduced compared to wild-type (WT) mice (Figure 1A). To further evaluate vascular development, we employed immunofluorescence staining to detect expression of the neonatal endothelial cell marker CD34 in placental tissue. Results showed a significant reduction in CD34-positive staining in the placenta of Coq4^−/−^ mice compared to WT mice, indicating markedly decreased neovascularization (Figure 1B).

### 3.2 Coq4 deficiency suppresses vascular endothelial cell migration and angiogenesis

**FIGURE 2:**
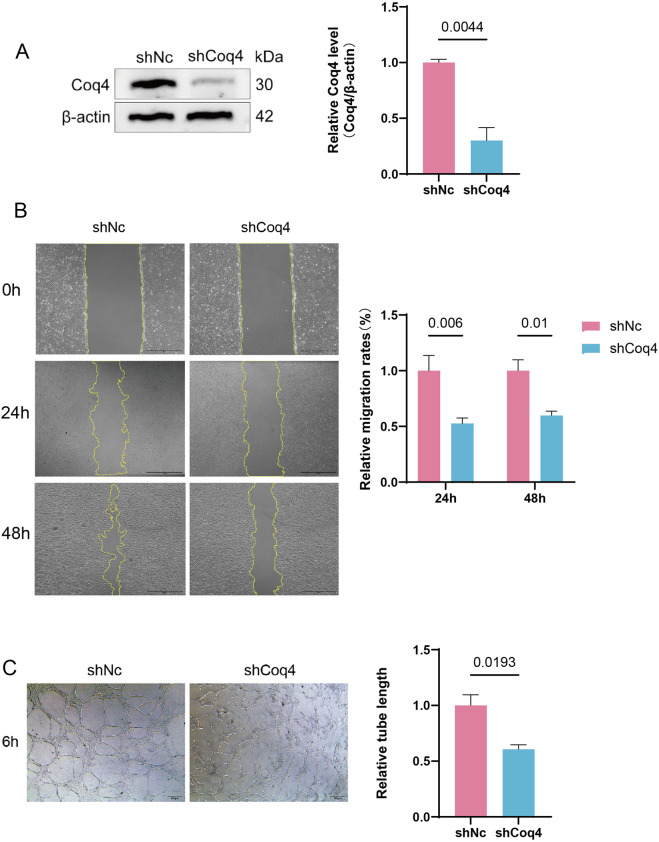
Coq4 deficiency suppresses vascular endothelial cell migration and angiogenesis. **(A)** Established stably transfected HUVEC cell lines with shNc and shCoq4, and the expression levels of Coq4 were detected by Western blotting, compared to shNc group, n = 3. **(B)** Cell scratch assay for detecting cell migration capacity and quantitative analysis, compared to shNc group, n = 3; **(C)** Matrigel tube formation assay for measuring angiogenesis capacity and quantitative analysis, compared to shNc group, n = 3.

Based on the above findings, to further investigate the specific mechanisms by which Coq4 functions in vascular development and homeostasis maintenance, we selected human umbilical vein endothelial cells (HUVECs) as an *in vitro* model for subsequent studies. First, we established stable Coq4 knockdown (shCoq4) and negative control (shNC) HUVEC cell lines using shRNA technology. Western blotting revealed significantly reduced Coq4 protein expression in the shCoq4 group compared to the shNC group (P < 0.01, Figure 2A), confirming successful establishment of the stable cell lines. Next, cell scratch assays evaluated migration capacity, revealing that Coq4 deficiency significantly inhibited cell migration (P < 0.01 at 24h; P < 0.05 at 48 h, Figure 2B). Simultaneously, the matrix gel formation assay evaluated angiogenesis capacity, revealing that Coq4 deletion significantly inhibited endothelial cell tube formation ability (P < 0.05, Figure 2C). These results collectively demonstrate that Coq4 deletion significantly suppresses both endothelial cell migration and tube formation capacity.

### 3.3 Transcriptome analysis of HUVECs with Coq4 knockdown

**FIGURE 3:**
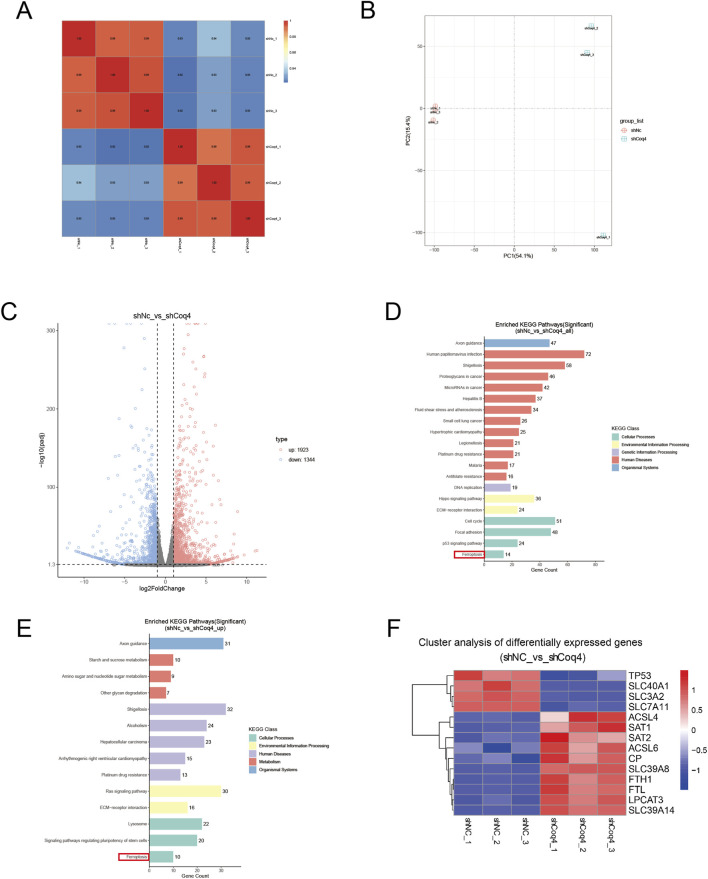
Transcriptome analysis of HUVECs with stabilized knockdown of Coq4. **(A)** Pearson correlation heatmap, where x-axis and y-axis represent the square of the correlation coefficient between samples. **(B)** Principal Component Analysis (PCA) plot, where x-axis represents the first principal component (54.1%) and y-axis represents the second principal component (15.4%). **(C)** Volcano plot of differentially expressed genes. The x-axis represents log2FoldChange, and the y-axis represents -log10 (padj). The two vertical dashed lines indicate the 2-fold expression difference threshold; the horizontal dashed line indicates the padj = 0.05 threshold. Red dots indicate genes upregulated in this combination, blue dots indicate genes downregulated in this group, and gray dots indicate genes with non-significant expression differences. **(D)** Top 20 significantly enriched pathways from KEGG analysis of differentially expressed genes, categorized by level 1 pathways. The x-axis shows the number of differentially expressed genes enriched in each pathway, while the y-axis displays different KEGG pathway names. **(E)** Significantly upregulated pathways from KEGG analysis of differentially expressed genes. **(F)** Significantly differentially expressed genes enriched in ferroptosis pathways. The x-axis represents samples, and the y-axis represents significantly differentially expressed genes.

Subsequently, we performed RNA-seq analysis on the established cell models. By calculating the FPKM values (fragments per kilobase per million mapped reads) for all genes in each sample, we obtained intra- and inter-group correlation coefficients and generated a correlation heatmap. Results showed that the intra-group correlation coefficient R^2^ exceeded 0.98 for biological replicates, while the inter-group correlation coefficient R^2^ between shCoq4 and shNc groups exceeded 0.90 (Figure 3A). To further validate intergroup differences and intragroup sample replication, principal component analysis (PCA) was performed. The PCA plot revealed significant dispersion between the shCoq4 and shNc groups while showing tight clustering within each group (Figure 3B). These results indicate significant intergroup differences, good intragroup sample reproducibility, and high data quality.

Differential gene expression analysis was performed using DESeq2 (threshold for differential gene screening: |log2FoldChange| > 1.0 and *P*adj < 0.05), and a volcano plot was generated. The results revealed that 1,923 genes were upregulated and 1,344 genes were downregulated in this cell model (Figure 3C). Subsequently, we categorized the top 20 significantly enriched pathways and identified ferroptosis pathways as significantly enriched, clearly classified as an upregulated pathway (Figures 3D,E). Subsequently, we analyzed significantly differentially expressed genes within the ferroptosis-enriched pathways. Compared to the shNc group, four genes were downregulated in the shCoq4 group, including key components of the anti-ferroptosis system Xc^−^: SLC7A11 and SLC3A2. Concurrently, ten genes were upregulated, including ferroptosis-promoting protein ACSL4 (Figure 3F).

These findings suggest at the transcriptomic level that Coq4 deficiency may promote ferroptosis in endothelial cells by modulating the expression of ferroptosis-related genes.

### 3.4 Coq4 deficiency induces ferroptosis in endothelial cells

**FIGURE 4:**
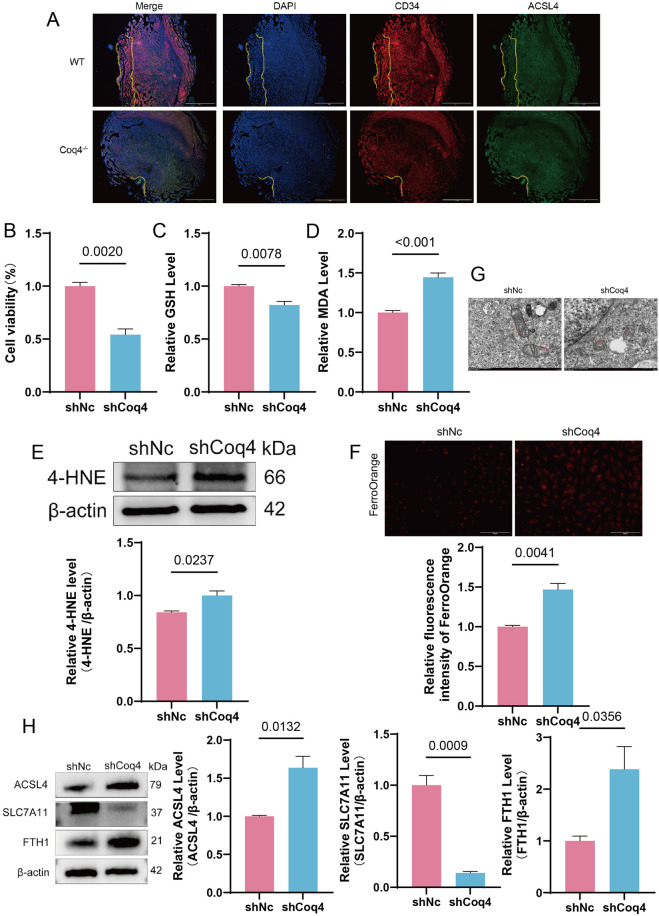
Coq4 deficiency induces ferroptosis in endothelial cells. **(A)** Immunofluorescence staining of placentas from WT and Coq4^−/−^ mice with DAPI (blue), CD34 (red), and ACSL4 (green). Scale bar = 750μm, n = 6. **(B)** CCK-8 assay for cell viability, compared to shNc, n = 6. **(C)** Commercial kit for GSH content detection, compared to shNc, n = 6. **(D)** MDA (malondialdehyde) levels measured by commercial kit, compared to shNc group, n = 6. **(E)** Western blotting for 4-HNE expression and quantitative analysis, compared to shNC group, n = 3. **(F)** FerroOrange assay for intracellular Fe^2+^, n = 3, Scale bar = 150 μm. **(G)** Mitochondrial morphology in shNc and shCoq4 cells under transmission electron microscopy, n = 3, Scale bar = 500 μm. **(H)** Western blotting for ACSL4, SLC7A11, and FTH1 protein expression levels and quantitative analysis, compared to shNc, n = 3.

To verify whether Coq4 deficiency indeed induces ferroptosis in vascular endothelial cells, we first conducted *in vivo* validation. Immunofluorescence results revealed that compared to WT mice, Coq4^−/−^ mice exhibited significantly elevated expression levels of ACSL4, a key regulator of ferroptosis, in placental endothelial cells (Figure 4A). We then further examined whether ferroptosis also occurred in the human umbilical vein endothelial cell model (shCoq4-HUVECs). First, the impact of Coq4 knockdown on cell viability was assessed. CCK-8 assay results revealed significantly reduced viability in the shCoq4 group compared to the shNc (*P* < 0.01, Figure 4B). Furthermore, intracellular levels of the primary antioxidant glutathione (GSH) were markedly decreased in the shCoq4 group relative to shNc (*P* < 0.01, Figure 4C), while lipid peroxidation products malondialdehyde (MDA) (*P* < 0.001, Figure 4D) and 4-hydroxy-2-nonenal (4-HNE) (*P* < 0.05, Figure 4E) were significantly elevated. FerroOrange probe detection revealed significant accumulation of Fe^2+^ in shCoq4 cells compared to shNc cells (Figure 4F). At the ultrastructural level, transmission electron microscopy (TEM) examination showed classical ferroptosis morphological features in the shCoq4 group, including reduced mitochondrial cristae, decreased mitochondrial volume, and increased membrane density, compared to the shNc group (Figure 4G). Additionally, Western blotting analysis revealed that compared to the shNc group, the shCoq4 group exhibited significantly upregulated expression of (ACSL4) (*P* < 0.05) and (FTH1) (*P* < 0.05). Conversely, expression of SLC7A11 was significantly downregulated (*P* < 0.001) (Figure 4H).

The above findings collectively reflect the hallmark features of ferroptosis, namely impaired antioxidant defense systems, disrupted iron metabolism, and accumulation of lipid peroxides. Therefore, we conclude that Coq4 deficiency induces ferroptosis in human endothelial cells, consistent with the bioinformatics analysis described above.

### 3.5 Coq4 knockdown induces ferroptosis in endothelial cells via the FSP1/CoQ10 axis but independently of GPX4

**FIGURE 5:**
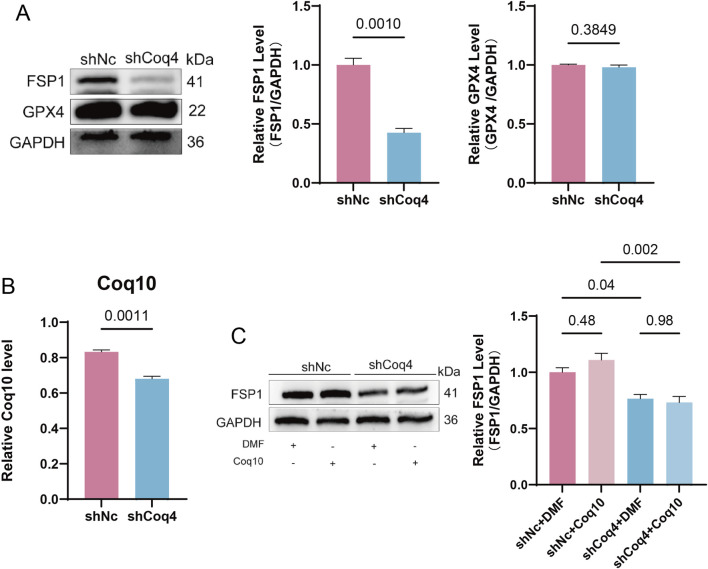
Coq4 Knockdown Induces Ferroptosis in Endothelial Cells via the FSP1/CoQ10 Axis but Independently of GPX4. **(A)** Expression levels of GPX4 and FSP1 were detected by Western blotting and quantitative analysis, compared to shNc, n = 3. **(B)** Quantitative analysis of intracellular CoQ10 levels using a detection kit, compared to shNc, n = 3. **(C)** Quantitative analysis of FSP1 protein expression following CoQ10 supplementation via Western blotting, n = 3.

In the anti-ferroptosis pathway, GPX4 and FSP1 exert parallel and independent roles (Yang et al., 2025). To investigate the specific mechanism by which Coq4 deficiency affects ferroptosis, we first assessed the protein expression levels of GPX4 and FSP1 in shCoq4-HUVECs. Western blotting revealed that although intracellular GSH levels were significantly reduced in shCoq4 cells compared to shNc cells, GPX4 protein expression showed no significant change, whereas FSP1 protein levels were markedly decreased (*P* < 0.01, Figure 5A). GSH serves as an essential cofactor for GPX4 enzymatic reactions, and its depletion typically inhibits GPX4 activity (Li et al., 2024; Ursini et al., 2020). However, in this study, GPX4 protein expression did not significantly change with GSH reduction, suggesting that ferroptosis induced by Coq4 knockdown may not rely on GPX4 protein degradation. The specific regulatory mechanism requires further elucidation. Subsequently, we focused on the FSP1 pathway. Given that the classical anti-ferroptosis pathway for FSP1 involves FSP1/CoQ10, and Coq4 is a key factor in CoQ10 biosynthesis, we further assessed CoQ10 levels. Results showed that intracellular CoQ10 levels were significantly reduced in the shCoq4 group compared to the shNc group (*P* < 0.01, Figure 5B). We therefore hypothesized that Coq4 knockdown may impair CoQ10 synthesis, leading to functional loss or reduced stability of its downstream effector FSP1 due to insufficient “substrate,” thereby weakening this anti-ferroptosis defense pathway parallel to GPX4. Therefore, we chose to restore CoQ10 to test this hypothesis. However, after CoQ10 supplementation, intracellular FSP1 protein levels in the shCoq4 group did not show significant recovery compared to the shNc group (Figure 5C). This indicates that FSP1 downregulation is not primarily caused by insufficient CoQ10 synthesis but is more likely a direct or indirect consequence of Coq4 deficiency.

### 3.6 FSP1 and CoQ10 synergistically improve ferroptosis induced by Coq4 deficiency

**FIGURE 6:**
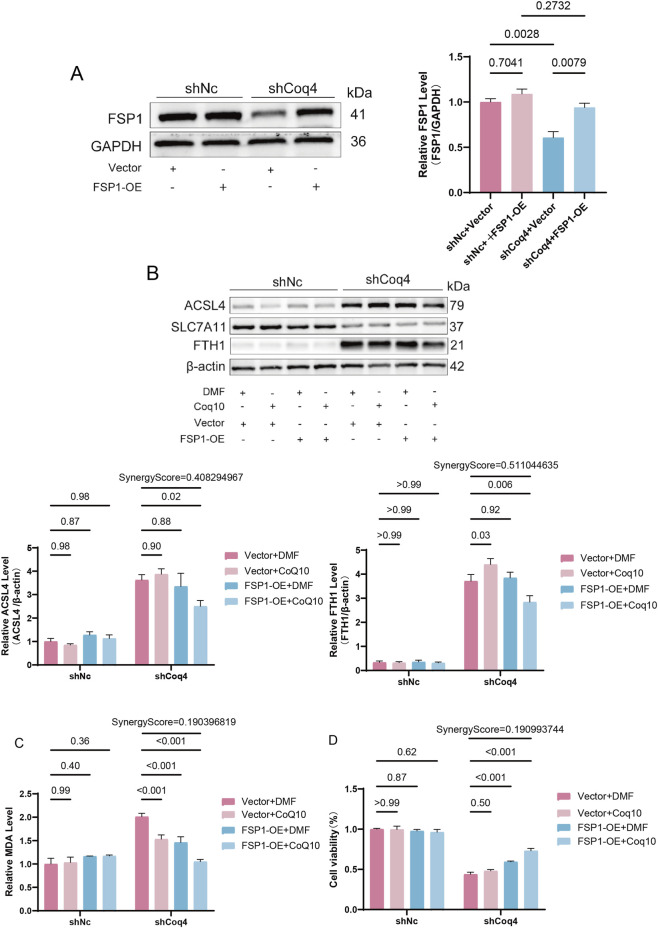
FSP1 and CoQ10 Synergistically Improve Ferroptosis Induced by Coq4 Deficiency. **(A)** Western blotting analysis of FSP1 overexpression and quantitative analysis, n = 3. **(B)** Western blotting analysis of ACSL4, SLC7A11, and FTH1 protein expression levels and quantitative analysis, n = 5. **(C)** Quantitative analysis of intracellular MDA (malondialdehyde) levels using a detection kit, n = 6. **(D)** Cell viability assay using a CCK-8 kit, n = 6.

Further experiments were conducted to assess the synergistic effects of FSP1 and CoQ10 in regulating ferroptosis through combined rescue strategies. First, we constructed an FSP1 overexpression (shCoq4+FSP1-OE) model based on shCoq4-HUVECs via plasmid expression. Western blotting revealed significantly elevated FSP1 protein levels in the shCoq4+FSP1-OE group compared to the empty vector control (shCoq4+Vector) (*P* < 0.01, Figure 6A), confirming successful model establishment. Subsequently, in the context of Coq4 knockdown, we performed a combined rescue experiment with FSP1 overexpression and CoQ10 supplementation. Western blotting analysis revealed that, compared to the control group (shCoq4+DMF+Vector), both ACSL4 and FTH1 expression were significantly downregulated in the treatment group (shCoq4+Coq10+FSP1-OE) (*P* < 0.05, Synergy Score > 0, Figure 6B), while SLC7A11 protein levels remained unchanged (Figure 6B). Concurrently, the treated group (shCoq4+CoQ10+FSP1-OE) exhibited significantly reduced levels of malondialdehyde (MDA), a lipid peroxidation product, (*P* < 0.001, Synergy Score > 0, Figure 6C), and showed significantly improved cell viability (*P* < 0.001, Synergy Score > 0, Figure 6D).

## 4 Discussion

This study integrates *in vivo* and *in vitro* experiments to reveal the critical role of Coq4 in placental vascular development. It demonstrates that Coq4 deficiency mediates ferroptosis in endothelial cells by regulating the FSP1-CoQ10 axis, thereby impairing normal vascular growth and formation. This discovery provides a novel perspective on the regulatory role of ferroptosis in physiological developmental processes. Endothelial cells not only constitute the structural framework of blood vessels during neovascularization and angiogenesis but also govern the establishment, remodeling, and functionalization of the embryonic vascular system through their differentiation, alignment, and signaling transduction (Roskoski, 2025). Previous studies by our group revealed that Coq4 deficiency impairs normal placental structural development, ultimately leading to embryonic lethality (He et al., 2024), though the precise mechanism remained unclear. This study further reveals that endothelial Coq4 deficiency disrupts normal placental vascular development in mice, accompanied by upregulation of ACSL4, a key ferroptosis marker. Such *in vivo* phenotypes suggest that Coq4 loss may induce endothelial ferroptosis, thereby disrupting normal vascular network formation, ultimately impairing placental structural development and causing embryonic lethality.

To gain deeper insights into the underlying molecular mechanisms, we selected HUVECs for subsequent *in vitro* experiments. First, we established a stable Coq4 knockdown cell line via lentiviral transfection (shCoq4-HUVECs). Transcriptomic analysis revealed elevated expression of ferroptosis pathways in this model, providing comprehensive gene expression evidence supporting our *in vivo* hypothesis and laying the foundation for further research. Additionally, we confirmed that Coq4 knockdown indeed induces ferroptosis in endothelial cells, as evidenced by intracellular glutathione (GSH) depletion, accumulation of lipid peroxides (MDA, 4-HNE), Fe^2+^ overload, typical mitochondrial morphological abnormalities, upregulation of ACSL4 and FTH1, and downregulation of SLC7A11, a key component of the anti-ferroptosis system Xc^−^. FTH1, a crucial ferritin subunit, maintains intracellular iron homeostasis by storing iron. Decreased expression or activity of FTH1 leads to the release of stored iron and increased free iron, which induces lipid peroxidation via the Fenton reaction and ultimately promotes ferroptosis (Tian et al., 2020; Tao et al., 2014; Sun et al., 2016). In this study, the observed trend of FTH1 upregulation may result from elevated intracellular Fe^2+^ levels, with compensatory increase in FTH1 expression to counteract ferroptosis.

Next, we examined two classic anti-ferroptosis pathways and found that despite a significant decrease in GSH levels, the protein expression of GPX4 remained largely unchanged, while FSP1 protein levels showed a marked reduction. GSH serves as an essential cofactor for GPX4 enzymatic reactions, and generally, GPX4 activity or function is closely linked to GSH levels (Toppo et al., 2009). However, other thiol-containing small molecules, such as cysteine and homocysteine, can also directly act as GPX4 substrates under specific conditions to inhibit ferroptosis (Xia et al., 2024). The precise regulatory mechanism underlying this phenomenon in our experiment requires further investigation.

Given that Coq4 is a key enzymatic factor in CoQ10 biosynthesis, we hypothesize that Coq4 deficiency disrupts CoQ10 synthesis, leading to functional loss or reduced stability of its downstream effector molecule FSP1 due to insufficient “substrate.” This, in turn, weakens the anti-ferroptosis defense pathway parallel to GPX4. However, FSP1 expression did not show significant improvement after CoQ10 supplementation. Therefore, we infer that the reduction in FSP1 is not a direct consequence of CoQ10 synthesis deficiency, but rather a direct or indirect result of Coq4 knockdown. This hypothesis requires further experimental validation.

To validate the role of the FSP1-CoQ10 axis, we conducted critical rescue experiments. Results revealed that simultaneous restoration of FSP1 and CoQ10 produced a potent synergistic effect, significantly ameliorating the ferroptosis phenotype induced by Coq4 knockdown. This improvement manifested as reduced ACSL4 and FTH1 protein expression, reduced lipid peroxide MDA levels, and enhanced cell viability. This effect can be interpreted as FSP1 and CoQ10 providing potent antioxidant capacity, directly counteracting oxidative stress and thereby improving the ferroptosis phenotype. As a concomitant or downstream feedback phenomenon of this reversal, levels of ferroptosis-promoting molecular markers (e.g., ACSL4) and compensatory markers (e.g., FTH1) also improved. Notably, SLC7A11 expression remained unaffected, robustly demonstrating that the FSP1-CoQ10 axis functions independently of the Xc-/GSH/GPX4 pathway (Doll et al., 2019). This finding not only aligns with Doll et al.'s pioneering theory that FSP1 quenches lipid peroxidation by reducing CoQ10 but also confers a distinct biological function to this pathway within the critical physiological process of vascular development. This effect specifically involves enhancing cellular antioxidant capacity and mitigating lipid peroxidation damage, thereby jointly maintaining endothelial cell homeostasis under oxidative stress.

Of course, this study also has certain limitations. First, we examined the protective effect of the Coq4-FSP1-CoQ10 axis against ferroptosis solely at the endothelial cell level. We did not investigate the therapeutic role of this axis in placental vascular development in Coq4 knockout mice, not did we validate its association with the injury mechanisms of adult cardiovascular diseases such as atherosclerosis and ischemia-reperfusion injury. Second, the specific molecular mechanisms by which Coq4 knockdown affects FSP1 protein levels require further investigation. Additionally, our exploration of Coq4 deficiency-induced placental dysfunction primarily focused on vascular and metabolic aspects, neglecting other potential implications. For instance, it remains unclear whether Coq4 deficiency impacts immune components at the maternal-fetal interface. Recent studies indicate that the residency and dysfunction of uterine immune cells also influence normal placental or fetal development (Ma et al., 2025b; Kang et al., 2024).

In summary, this study demonstrates that the Coq4-FSP1-CoQ10 pathway—independent of GPX4—plays an indispensable role in embryonic placental vascular development. It further reveals that CoQ10 supplementation alone is insufficient to mitigate Coq4-knockdown-induced ferroptosis, while simultaneous supplementation of CoQ10 and FSP1 more effectively mitigates ferroptosis. This provides partial rationale and therapeutic direction for the limited efficacy of CoQ10 monotherapy in patients with CoQ10 deficiency caused by Coq4 or other CoQ10 synthase mutations. This not only deepens our understanding of vascular biology and the complexity of ferroptosis regulatory networks, but also offers new insights into the pathogenesis of pregnancy complications triggered by placental dysfunction and ferroptosis-related vascular diseases, while offering novel approaches for exploring potential therapeutic targets.

## Data Availability

The datasets presented in this study can be found in online repositories. The names of the repository/repositories and accession number(s) can be found in the article/supplementary material.
